# TRK Inhibitors: Tissue-Agnostic Anti-Cancer Drugs

**DOI:** 10.3390/ph14070632

**Published:** 2021-06-29

**Authors:** Sun-Young Han

**Affiliations:** Research Institute of Pharmaceutical Sciences and College of Pharmacy, Gyeongsang National University, Jinju-si 52828, Korea; syhan@gnu.ac.kr

**Keywords:** Trk, NTRK, tissue-agnostic, larotrectinib, entrectinib, Trk fusion

## Abstract

Recently, two tropomycin receptor kinase (Trk) inhibitors, larotrectinib and entrectinib, have been approved for Trk fusion-positive cancer patients. Clinical trials for larotrectinib and entrectinib were performed with patients selected based on the presence of Trk fusion, regardless of cancer type. This unique approach, called tissue-agnostic development, expedited the process of Trk inhibitor development. In the present review, the development processes of larotrectinib and entrectinib have been described, along with discussion on other Trk inhibitors currently in clinical trials. The on-target effects of Trk inhibitors in Trk signaling exhibit adverse effects on the central nervous system, such as withdrawal pain, weight gain, and dizziness. A next generation sequencing-based method has been approved for companion diagnostics of larotrectinib, which can detect various types of Trk fusions in tumor samples. With the adoption of the tissue-agnostic approach, the development of Trk inhibitors has been accelerated.

## 1. Introduction

**Figure 1:**
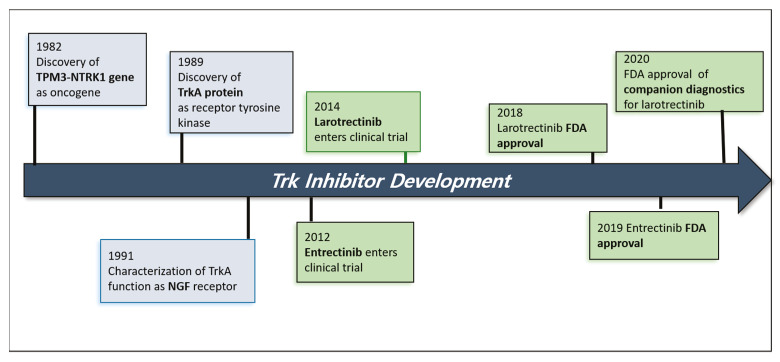
Timeline for the clinical development of larotrectinib and entrectinib. TPM3, tropomyosin 3; NTRK1, neurotrophic tyrosine receptor kinase 1; TrkA, tropomyosin receptor kinase A; NGF, nerve growth factor; FDA, US Food and Drug Administration.

Tropomyosin receptor kinases (Trk) are tyrosine kinases encoded by neurotrophic tyrosine/tropomyosin receptor kinase (NTRK) genes [1]. Chromosomal rearrangement of NTRK genes is found in cancer tissues [2]. The resulting fusion proteins containing part of the Trk protein have a constitutively active form of kinase that transduces deregulating signals. There is active progress in the development of small molecule inhibitors against Trk kinases in the field of cancer therapeutics [1]. Currently, larotrectinib and entrectinib are two approved drugs for Trk fusion-positive cancers in the market [3,4]. The timeline for the clinical development of the two Trk inhibitors is shown in Figure 1.

A unique process of drug development, known as tissue-agnostic development, was employed for larotrectinib and entrectinib approval. Patients for tissue-agnostic clinical trials were selected based on the presence of NTRK gene rearrangement, independent of tumor type [5,6]. Trk fusion-positive tumors of several cancer types were tested for Trk inhibitors, and excellent efficacy of these drugs was shown in tissue-agnostic trials.

In this review, the development process and pharmacological efficacy of current Trk inhibitors in the market will be described, along with some discussion on the Trk inhibitors currently in clinical development. In addition, we will review the development process of tissue-agnostic drugs. Finally, we aim to provide perspectives learned from the pioneering approach of tissue-agnostic therapy for Trk inhibitors.

## 2. Tissue-Agnostic Drug Development

Targeted cancer therapies that act on specific molecules have become mainstream strategies for anti-cancer drug development. There are two milestones for targeted cancer drug development: trastuzumab and imatinib. Trastuzumab is a monoclonal antibody specific to the human epidermal growth factor receptor 2 (HER2) protein [7]. The US Food and Drug Administration (FDA) approval for trastuzumab was obtained in 1998 for metastatic breast cancer overexpressing HER2 protein [8]. Imatinib is a small-molecule drug targeting the fusion protein BCR-ABL. The fusion protein is generated by chromosomal rearrangement in chronic myeloid leukemia cells. The remarkable efficacy of these two drugs paved the way for the era of targeted cancer therapy, and this made kinase family proteins major targets for cancer therapy [9].

The concept of targeted therapy expanded to the term precision medicine, personalized medicine, or stratified medicine, meaning “targeting drugs for each genetic profile” [7]. In contrast to the traditional “one-size-fits-all” approach, individualizing pharmacotherapy was emphasized upon due to the factors of disease heterogeneity and genetic variability [10]. Biomarkers that can predict therapeutic responses are important elements in precision medicine. Therefore, the diagnosis of biomarkers has become an important step in precision medicine, generating new terms such as companion diagnostics (CDx) or drug-diagnostic co-development [7]. With the adoption of CDx, it was possible to enroll only selected patients who were likely to respond to drug therapy. Clinical research involving a relatively small number of patients, enabled by screening out of non-responders, is called enrichment trial [11]. Trastuzumab was the first drug developed using a CDx approach. A diagnostic assay (HercepTest), which tests the expression of HER2 in breast tumors was developed and approved together with the drug [12]. Another representative example of CDx and enrichment trials is crizotinib, an anaplastic lymphoma kinase (ALK) inhibitor for non-small cell lung cancer (NSCLC) patients [13]. ALK fusion proteins caused by chromosomal rearrangement are found in approximately 4% of NSCLC patients, and these ALK fusion proteins have been reported to induce tumorigenesis. Enrichment clinical trials were conducted for crizotinib development in ALK fusion-positive NSCLC patients, and the number of patients in phase I trials was only 143. Diagnostic tests for ALK gene rearrangement were developed in conjunction with crizotinib development, and the approval of drugs and diagnostic tests were linked and included in the drug labeling.

Before the concept of tissue-agnostic drug was introduced, the development process of precision medicine included only one type of tumor. In the case of crizotinib, only NSCLC patients were included in the clinical trials, even though ALK gene fusion was originally found in anaplastic large cell lymphoma (ALCL) as well [14]. ALK translocation has also been discovered in rare tumors called inflammatory myofibroblastic tumors (IMTs) [15]. Clinical trials of crizotinib for patients with ALCL are ongoing. If clinical research was implemented regardless of tumor type, crizotinib could be used in ALCL and IMT patients as well as in subsets of NSCLC patients. In this way, clinical research with patient selection based on molecular features would have benefitted more cancer patients.

Therefore, a biomarker-guided drug development process has been proposed and successfully applied to three FDA-approved drugs. The immune checkpoint inhibitor, pembrolizumab, and two Trk inhibitors, larotrectinib and entrectinib, underwent tissue-agnostic development. Tissue-agnostic drugs target specific genetic molecular features regardless of tumor sites [16]. Terms such as histology-agnostic, tumor-agnostic, site-agnostic, pan-tumor therapies are used, depending on the literature [17]. If specific genetic aberrations are found across several tumor types, tissue-agnostic drug development can be utilized. Select ongoing tumor-agnostic developments with several cancer targets are listed in Table 1.

As a type of clinical research, encompassing different tumor types with the same molecular features is called a basket trial. Unlike enrichment trial, which generally consists of patients with a single tumor type, in basket trials, patients are selected based on their molecular characteristics, regardless of tumor histology. Sometimes, basket trials are viewed as a set of sub-trials [27]. Hypothetically, if crizotinib is developed using a basket trial, the basket trial would be composed of sub-trial 1 with NSCLC, sub-trial 2 with ALCL, and sub-trial 3 with IMT, all with ALK fusion-positive tumors. The results will be analyzed either by tumor type within the sub-trials or altogether.

Traditional clinical trials are based on randomization in new treatment vs. standard of care to avoid selection bias. With the introduction of targeted therapy, molecular segmentation of cancer resulted in a small patient population. And this became a challenge for conducting clinical trials [28]. In crizotinib phase 3 clinical trials in Europe, for example, there was a patient selection process from NSCLC patients. A total of 4967 NSCLC patients were screened, and 347 ALK fusion-positive patients were selected and randomized. The clinical benefit of crizotinib over chemotherapy was shown with overall response rates (65% vs. 20%) and a median PFS (7.7 months vs. 3 months) [29]. Given the large number of patients to be screened and the high overall response rate, the requirement of randomization was called into question. With the introduction of drug development in a tissue-agnostic way, FDA approval could be granted based on the nonrandomized trials. Pembrolizumab obtained FDA approval based on the clinical trials with 149 patients [30], larotrectinib with 55 patients [3], and entrectinib with 54 patients [4]. Given the extremely low prevalence of NTRK fusion (0.31%) [31], it would take a much more extended period to recruit patients for randomized clinical trials.

Tumor-agnostic approach cannot be adopted for all oncogenic alterations [6,16]. The B rapidly accelerated fibrosarcoma (BRAF) inhibitor vemurafenib is very effective in melanoma and NSCLC patients with the BRAF V600 mutation, an activating mutation of BRAF. However, only 5% of colorectal cancers harboring the BRAF V600 mutation respond to vemurafenib therapy [32]. Several clinical studies have been conducted on trastuzumab for tumors with HER2 mutations or amplification; the clinical benefits differed depending on the tumor type. A subset of colorectal cancer with HER2 amplification (5%) showed an overall response rate (ORR) of 30% only for lapatinib plus trastuzumab therapy [33]. Despite the 20 years of clinical research on various tumor types with HER2 aberration, the indications for trastuzumab are only breast cancer and gastroesophageal cancer. These studies clearly show that not all biomarkers can be developed in a tissue-agnostic manner. Besides Trk and PD-1 inhibitors already approved by the FDA, the targets for potential tissue-agnostic drugs in clinical development are ret proto-oncogene (RET), ALK, fibroblast growth factor receptor (FGFR), Axl, ros proto-oncogene 1 (ROS1), and BRAF (Table 1) [17]. It is interesting to note that oncogenic alterations caused by chromosomal rearrangements, RET, ALK, FGFR, and ROS1 fusion proteins account for the majority of cancer targets in tissue-agnostic therapy.

Besides regulatory reasons, there are several factors why drug development processes have been restricted to one cancer type before pembrolizumab. There are different available therapies and unmet medical needs for each tumor type, factors that are considered substantially for development decisions and drug approval. In addition, the endpoints of drug efficacy for each tumor type are different. As some drug discovery experts term as ‘low hanging fruit’, generally a tumor type with no known therapy and urgent unmet medical need is first taken up for clinical research, subsequently followed by development in another tumor type.

With the introduction of tissue-agnostic drug development approaches, several tumor types can be subjected to clinical trials at the same time. Tissue-agnostic drug development is also good news for rare cancer patients. Due to the small number of patients, it is not easy to conduct clinical trials for cancers with low incidence. Tissue-agnostic drug development enables the participation of rare cancer patients in clinical trials; therefore, rare cancer patients can benefit from this new paradigm of drug approval process [28].

## 3. Trk Inhibitors

### 3.1. Trk and Cancer

The Trk family is comprised of three isoforms, TrkA, TrkB, and TrkC, encoded by NTRK1, NTRK2, and NTRK3, respectively. The Trk family is abundantly expressed in the nervous system. Ligands for Trk cell surface receptor tyrosine kinase are nerve growth factor (NGF) for TrkA, brain-derived neurotropic factor or neurotropin 4 for TrkB, and neurotropin 3 for TrkC [1]. Downstream signaling for Trk receptor kinases is primarily mediated by the phospholipase Cγ, mitogen-activated protein kinase, and phosphoinositol-3 kinase pathways.

As implicated by the expression pattern and cognate ligands, neuronal development and differentiation have been reported as major functions of Trk pathways. The importance of TrkA in neuronal development is shown in case of genetic diseases with loss-of-function NTRK genes. Hereditary disorder called congenital insensitivity to pain (CIPA) is reported to have NTRK1 gene mutations [34]. The absence of TrkA during fetal development results in the loss of pain sensing in TrkA-deficient mice [35,36], suggesting the crucial role of TrkA signaling in nociceptive reception [37]. In case of TrkB, impairment of TrkB signaling causes hyperphagia and consequent obesity [38].

Various mechanisms of Trk activation exist in cancer, including somatic mutations, activating splice variants, Trk overexpression, and NTRK fusion [1]. The most common mechanism of Trk activation in cancer is fusion involving NTRK1, NTRK2, and NTRK3. Trk fusion proteins are generated by chromosomal rearrangements between NTRK genes, including the kinase domain, with different partner genes. The resulting fusion proteins are chimeras with a constitutively activated Trk kinase, independent of ligand binding [39].

The first identified NTRK fusion was tropomyosin 3 (TPM3)-NTRK1, which was found in patients with colorectal cancer [40]. Subsequently, Trk fusion proteins with different partners have been identified in a variety of cancer types. The NTRK fusions include translocated promoter region (TPR)-NTRK1 in thyroid cancer [41], tripartite motif containing 24 (TRIM24)-NTRK2 [42] and ETS variant transcription factor 6 (ETV6)-NTRK3 in fibrosarcoma [43].

### 3.2. Larotrectinib

Larotrectinib, also known as ARRY-470, LOXO-101, and Vitrakvi^®^, is the first FDA-approved Trk inhibitor with high potency and selectivity. Larotrectinib inhibits the in vitro kinase activity of TrkA by blocking ATP-binding sites with an half maximal inhibitory concentration (IC_50_) of 10 nM [44]. Kinase selectivity analyses with 226 kinases indicated that larotrectinib is highly selective for TrkA, TrkB, and TrkC. Except for one kinase, TNK2, inhibition of no other notable kinases was observed. Larotrectinib potently suppressed the growth of cancer cells harboring TrkA and TrkB fusion proteins in vitro and in vivo [45].

Based on the impressive preclinical efficacy, clinical trials of larotrectinib started in 2014. Approval of larotrectinib is based on three clinical studies: an adult phase 1 trial (NCT02122913; LOXO-TRK-14001), a pediatric phase 1/2 trial called SCOUT (NCT02637687; LOXO-TRK-15003), and an adult/adolescent phase 2 basket trial called NAVIGATE (NCT02576431; LOXO-TRK-15002). Five journal articles have been published to date on these clinical trials. Deobele et al. described a case of a patient with soft-tissue sarcoma treated with larotrectinib in the LOXO-TRK-14001 trial [45]. Cases of five patients in the SCOUT clinical trial were discussed in the paper by Dubois et al. [46], and the overall phase 1 study results of the SCOUT trial with 24 pediatric solid tumor patients were published by Laetsch et al. [47]. The combined analyses of the three clinical trials stated above (LOXO-TRK-14001, SCOUT, and NAVIGATE) were published for 55 patients from 2015 to 2017 (data cut-off), and larotrectinib was approved on the basis of these results [48]. Clinical research continued, and data from 2014 to 2019 with 159 patients were analyzed and reported in 2020 by Hong et al. [49].

According to a recent report by Hong et al. [49], 159 patients with Trk fusion-positive cancers were treated with larotrectinib, with ages ranging from less than 1 month to 84 years. There were 153 evaluable patients, and the ORR was 79% (121 patients), consisting of complete response in 16% (24 patients) and partial response in 63% (97 patients). More than 16 tumor types were included in the clinical research, and clinical benefits were observed in a wide range of tumor types indicating tumor-agnostic activity. Trk fusions for NTRK1, NTRK2, and NTRK3 were included with 29 distinct fusion partners. The response rate was independent of the Trk subtype and upstream fusion partners. The adverse events of larotrectinib treatment were predominantly grade 1 and 2, indicating that long-term administration is feasible.

### 3.3. Entrectinib

Entrectinib, also called RXDX-101, NMS-E628, and Rozlyreck^®^, is an orally available inhibitor of TrkA/B/C, ROS1, and ALK [4]. Potent in vitro kinase activity for TrkA/B/C, ROS1, and ALK exhibited IC_50_ values between 1 nM and 12 nM [50]. The growth of cancer cell lines addicted to these kinases was suppressed upon entrectinib treatment in vitro and in vivo. Entrectinib was designed to have intracranial activity; thus, penetration into the central nervous system (CNS) has been demonstrated in preclinical models. The brain/plasma ratio of entrectinib in mice was 0.43 [50].

Three representative clinical trials of entrectinib are ALKA-372-001 (EudraCT 2012-000148-88), STARTRK-1 (NCT02097810), and STARTRK-2 (NCT02568267). ALKA-372-001 and STARTRK-1 are phase 1 dose-escalation studies, while STARTRK-2 is a phase 2 basket trial. The interim results of ALKA-372-001 and STARTRK-1 in 119 patients were published in 2017 [51]. Integrated analyses of the three clinical trials were reported in 2020 [52]. Another key trial is STARTRK-NG (NCT02650401), which is a phase 1/1b multicenter, dose-escalation study in patients aged 2–21 years with recurrent or refractory solid tumors and primary CNS tumors [4]. The STARTRK-1, -2, and -NG trials are ongoing.

Pooled analyses of ALKA-372-001, STARTRK-1, and STARTRK-2 with a data cut-off date in May 2018 were performed [52]. Efficacy-evaluable patients included 54 adults with NTRK fusion-positive solid tumors. Ten different types of tumor types were included, with the predominant types being sarcoma (13 (24%) patients) and NSCLC (10 (19%)). Among the 54 patients, 12 (22%) had baseline CNS disease and 31 (57%) had an objective response, comprising of 4 (7%) complete responses and 27 (50%) partial responses. 9 patients (17%) showed stable disease. The median response duration was 10 months. Among the 11 patients with brain metastases at baseline, six patients had measurable disease for intracranial response: four with complete response or partial response, one with stable disease, and one with progressive disease. The overall safety-evaluable population was 355 patients, and the most common grade 3 or 4 adverse events were weight gain and anemia. The most serious adverse events were nervous system disorders, reported in 10 (3%) of the 355 patients. Overall, entrectinib achieved anti-tumor activity against tumors harboring NTRK1, NTRK2, and NTRK3 fusions, including CNS activity.

### 3.4. Trk Inhibitors in Clinical Development

Other Trk inhibitors have been developed to overcome resistance mutations. Selitrectinib (LOXO-195, BAY 2731954) and repotrectinib (TPX-0005) are next-generation Trk inhibitors with efficacy against Trk with acquired resistance. Resistance mutations in the amino acid substitution of the Trk kinase domain have been reported in clinical cases [53]. The most common mutations are “solvent-front” mutations, termed after the hydrophilic solvent-exposed portion of the kinase domain ATP-binding site. TrkA G595R and TrkC G623R are solvent-front mutations, and TrkB mutations have not been reported to date [1]. In addition, gatekeeper mutations (TrkA F589L) and xDFG (Aspartate-Phenylalanine-Glycine) site mutations of TrkA (G667S) and TrkC (G696A) were identified in patients.

Selitrectinib has been developed in parallel with clinical trials to prepare for the emergence of resistance to larotrectinib [54]. Selitrectinib showed potent activity against TrkA/C solvent-front- and xDFG site-mutated forms as well as TrkA/C wild-type in in vitro and in vivo xenograft experiments. Kinase profiling of selitrectinib showed that it is highly selective for Trk kinase. A Phase1/2 clinical trial for selitrectinib is ongoing (NCT03215511), and interim results have been reported [20]. Patient selection was based on presence of tumor with TRK fusion and tumor progression or intolerance to prior Trk inhibitors. A total of 31 patients were analyzed and an ORR of 34% was reported. Trk mutations were identified in 20 patients, including 14 solvent-front, 4 gatekeeper, and 2 xDFG mutations, and a complete or partial response was observed in 9 patients.

Repotrectinib is a next-generation TKI inhibitor designed to inhibit the solvent-front mutations of Trk, ROS1, and ALK. In addition to TrkA G595R and TrkC G623R, repotrectinib is also active against solvent-front mutations of ROS1 and ALK in vitro and in vivo [18]. A phase 1/2 clinical trial of repotrectinib (TRIDENT-1, NCT03093116) is ongoing for TKI-refractory patients.

### 3.5. Adverse Effects of Trk Inhibitors on the Central Nervous System

Given the physiological roles of Trk signaling in the neuronal system, the effects of Trk inhibitors in the CNS are expected. It is reasonable to expect and prepare for the adverse effects of Trk inhibitors on the CNS. As described above, congenital insensitivity to pain is caused by NTRK1 mutation [34]. Based on the role of NTRK1 in pain sensing, NGF/TrkA is a target for analgesics, and several small molecules and antibodies modulating the NGF/TrkA pathway are under development [55].

Trk signaling is known to play a role in nerve growth during the fetal period, while NGF function is reported to induce pain in adulthood [55]. From the beginning of Trk inhibitor development, there was a concern for adverse CNS effects, and thus, adverse events were closely observed and characterized [56]. Representative adverse events related to neurological systems include withdrawal pain, weight gain, and dizziness [48,52].

Patients who discontinued Trk inhibitor therapy experienced symptoms of pain. Full-body ache, muscle pain, allodynia, and concurrent flares of pre-existing pain are described as withdrawal symptoms [56]. The mechanisms of withdrawal pain are not clear, but it is presumed to be caused by increased expression of transient receptor potential vanilloid I, a nociceptive mediator [57]. Weight gain was observed in more than 50% of patients, as expected from the role of the brain-derived neurotropic factor (BDNF)-TrkB pathway in appetite centers [38,58,59]. Hyperphagia and consequent obesity were observed upon BDNF-TrkB axis impairment in both mice and humans. Dizziness was caused upon TrkB and TrkC inhibition. Mutant mice with low levels of BDNF in the cerebellum developed ataxia [60]. Mice with NTRK3 gene knockout exhibited abnormal movement and posture [61]. These adverse events in the CNS caused by on-target inhibition of Trk inhibitors should be monitored carefully.

### 3.6. Identification of NTRK Fusions

Obviously, the diagnostic identification of NTRK fusion genes is an important process in tissue-agnostic Trk inhibitor development. Patient selection with NTRK fusion-positive cancers is a key point in screening responders and non-responders. NTRK fusions can be evaluated using immunohistochemistry (IHC), fluorescence in situ hybridization (FISH), reverse transcriptase polymerase chain reaction (RT-PCR), and next-generation sequencing (NGS) [2].

IHC using antibodies against Trk proteins can be utilized for identification of NTRK fusion, as Trk proteins are poorly expressed in normal adult tissues [62]. Positive staining in the IHC test can be interpreted as the presence of NTRK fusions. Information about cellular localization of fused proteins can also be obtained from IHC results. The localization of fusion proteins depends on the normal localization of the fusion partner [62]. For example, LMNA-NTRK1 fusion result in nuclear membrane staining due to the nuclear membrane protein lamin A/C encoded by the LMNA gene, while ETV6-NTRK3 fusions exhibit nuclear staining due to the ETV6-encoding protein located in the nucleus. IHC can be used for diagnosis in conjunction with other diagnostic methods.

FISH is a highly sensitive and specific tool for the detection of fused genes generated from chromosomal rearrangements, such as ALK, ROS1, and RET. In general, FISH has many advantages, such as high sensitivity and quick turn-around time. In case of NTRK1, 2, and 3 fusions, three separate assays are required for each gene. When chromosomal rearrangements involve non-canonical sites or intra-chromosomal rearrangements, FISH can lead to false negative results [63].

RT-PCR uses primers recognizing the 5′-fusion partner and NTRK kinase domain. Since there are numerous fusion partners, RT-PCR has limitations in clinical applications. Furthermore, fused genes with novel fusion partners cannot be detected using RT-PCR [64].

NGS offers the advantage of simultaneous assessment of multiple oncogenes. NGS is a highly sensitive and specific assay via which unknown NTRK fusions can be identified. DNA- and RNA-based NGS assays are currently available. Sometimes DNA-based NGS can fail to detect NTRK fusions because of the large intronic regions. RNA-based NGS assays can overcome this disadvantage of DNA-based NGS, as the results are not affected by intron size. However, the unstable nature of RNA is a major limitation of this assay. Currently, NGS assay sequencing of mature mRNA is considered the gold standard for NTRK fusion detection [64].

In 1998, the FDA approved the first CDx for drug-diagnostic co-development, HercepTest for trastuzumab therapy [65]. According to the regulatory guidance issued by the FDA in 2014, CDx testing is mandatory and must be performed before the use of the corresponding therapeutic product [66]. The FDA has approved an NGS-based CDx test for larotrectinib (Foundation Medicine Inc., F1CDx) [65]. It is a DNA-based NGS assay and was approved based on the retrospective testing of available tumor samples from patients in the three clinical trials of larotrectinib described above. Currently, there is no FDA-approved CDx for entrectinib.

## 4. Conclusions

Novel Trk inhibitors, larotrectinib and entrectinib, exhibit impressive clinical activity in cancer patients with Trk fusions. The tissue-agnostic drug development approach made it possible for a relatively efficient clinical development process. Although the tissue-agnostic approach cannot be applied to all cancer targets, these processes can expedite some of the drug development projects with unique biomarkers that enable patient selection. The adoption of the tissue-agnostic approach is expected to increase, resulting in an accelerated development process and the possibility of developing therapy for rare cancers.

## Figures and Tables

**Table 1:** Select tissue-agnostic developments in clinical trials.

Drug Name	Target	Development Phase	Reference
Repotrectinib (TPX-0005)	Trk/ALK, ROS1	II	[18,19]
Selitrectinib (LOXO-195)	Trk	II	[20]
Taletrectinib (DS-6051b)	Trk/ROS1	I	[21]
TPX-0046	RET/SRC	II	[22]
Debio1347	FGFR	II	[23]
Dubermatinib (TP-0903)	AXL	I	[24]
PLX8394	BRAF	II	[25]
Tislelizumab (BGB-A317)	PD-1	II	[26]

ALK, anaplastic lymphoma kinase; ROS1, c-ros proto-oncogene 1; RET, ret proto-oncogene; SRC, src proto-oncogene; FGFR, fibroblast growth factor receptor; BRAF, B rapidly accelerated fibrosarcoma; PD-1, programmed cell death protein 1.

## Data Availability

Data sharing not applicable.

## Abbreviations

ABL	abl proto-oncogene
ALCL	anaplastic large cell lymphoma
ALK	anaplastic lymphoma kinase
BCR	breakpoint cluster region
BDNF	brain-derived neurotrophic factor
BRAF	B rapidly accelerated fibrosarcoma
CDx	companion diagnostics
CIPA	congenital insensitivity to pain with anhidrosis
CNS	central nervous system
ETV6	ETS variant transcription factor 6
FDA	food and drug administration
FGFR	fibroblast growth factor receptor
FISH	fluorescence in situ hybridization
HER2	human epidermal growth factor receptor 2
IC_50_	half maximal inhibitory concentration
IHC	immunohistochemistry
IMT	inflammatory myofibroblastic tumor
LMNA	lamin A/C
NGF	nerve growth factor
NGS	next-generation sequencing
NSCLC	non-small cell lung cancer
NTRK	neurotrophic tyrosine receptor kinase
ORR	overall response rate
PD-1	programmed cell death protein 1
RET	ret proto-oncogene
ROS1	c-ros proto-oncogene
RT-PCR	reverse transcriptase polymerase chain reaction
SRC	src proto-oncogene
TNK2	tyrosine kinase non receptor 2
TPM3	tropomyosin 3
TPR	translocated promoter region
TRIM24	tripartite motif containing 24
Trk	tropomyosin receptor kinase
xDFG	Aspartate-Phenylalanine-Glycine
